# Ideal Clinic Realisation and Maintenance programme implementation in rural KwaZulu-Natal

**DOI:** 10.4102/phcfm.v16i1.4586

**Published:** 2024-10-09

**Authors:** Donald T. Mhlungu, Geertien C. Boersema, Mokholelana M. Ramukumba

**Affiliations:** 1 Department of Health Studies, College of Human Sciences, University of South Africa, Pretoria, South Africa

**Keywords:** Ideal Clinic Realisation and Maintenance programme, Ideal Clinic, implementation, KwaZulu-Natal, primary healthcare, professional nurse, rural

## Abstract

**Background:**

The delivery of quality primary healthcare (PHC) services is vital for enhancing the health status of rural communities, yet persistent barriers exist in resource-constrained rural settings.

**Aim:**

The study explored perspectives on the barriers to and facilitators of implementing the Ideal Clinic Realisation and Maintenance (ICRM) programme as a quality assurance initiative in a rural KwaZulu-Natal subdistrict.

**Setting:**

Professional nurses and healthcare managers from seven PHC clinics in a rural subdistrict of KwaZulu-Natal and supervising managers from a district hospital participated in this study.

**Methods:**

Telephonic semi-structured interviews were conducted using a qualitative case study approach with the purposively selected sample. Data were inductively and thematically analysed.

**Results:**

Themes included ICRM programme organisation, barriers and facilitators for implementing the ICRM programme. Barriers in rural PHC settings included overburdened clinics, suboptimal infrastructure, staff burnout, poor communication and non-adherence to clinical guidelines. Despite obstacles, programme implementation was facilitated through stakeholder support and teamwork. Participants emphasised the need for infrastructure upgrades, more human and physical resources, and maintenance of stakeholder support.

**Conclusion:**

If challenges are mitigated and supportive factors are leveraged, the potential for successful programme implementation and improved healthcare delivery can benefit both healthcare providers and recipients.

**Contribution:**

Through providing insight into the perspectives of both implementers and supervisors, the study informs stakeholders and policymakers about difficulties encountered and potential improvements to be made in the implementation of the ICRM programme in rural PHC.

## Introduction

Accessibility to quality primary healthcare (PHC) services globally, particularly in rural areas, has become a significant concern because of rapid population growth. In sub-Saharan regions, problems encountered in rural areas are shortages of skilled staff, poor accessibility, budgetary constraints and inadequate infrastructure, leading to suboptimal healthcare quality.^1^ Recognising this, the South African government embraced a PHC approach as a strategic initiative to fulfil healthcare needs in rural communities. South Africa, as a developing country with many rural communities dependent on PHC, faces numerous obstacles relating to the provision of healthcare, with 43% of deaths attributed to non-communicable diseases, 34% to human immunodeficiency virus (HIV) and tuberculosis (TB), 14% to other communicable diseases and 10% to injuries.^2,3,4^

Providing quality healthcare to fulfil the diversity of healthcare needs requires quality improvement programmes. The Ideal Clinic Realisation and Maintenance (ICRM) programme was introduced in 2013 to achieve quality improvement in South Africa to overcome shortcomings and prepare healthcare facilities to implement the intended national health insurance.^5^ Despite these efforts, PHC service providers in the selected rural subdistrict in KwaZulu-Natal and other parts of the country were not performing as expected in relation to some aspects of the ICRM programme, with, for example, non-adherence to guidelines on archiving and filing of patients’ records, slow increase of immunisation coverage of babies, a lack of essential equipment and more being observed.^6,7^ The primary objective of the ICRM programme is to improve the quality of patient care at PHC level through better monitoring and evaluation.^5,8^ The programme is implemented according to the Ideal Clinic dashboard consisting of 238 elements, 10 components and 32 subcomponents. The 10 components are administration, integrated clinical service management (ICSM), medicine supplies and laboratory services, human resources, support services (e.g. security), infrastructure, health information management, communication, the district health system support and implementing stakeholders.^9^ To allow for a systematic change process, each element within the components is colour-coded, indicating the degree of improvement needed; fully functional elements are coded green, those that require further work are coded orange, and elements coded red indicate the absence or a lack of functioning.^9^ In addition, the elements are categorised as vital, essential or important. On the basis of the average score obtained during formative or summative assessment of specific elements, Ideal Clinic status is further classified as silver, gold or platinum.^5^

A PHC clinic must obtain 100% for non-negotiable vital elements, 70% for essential elements and 69% for important elements. Elements are scored according to the performance of the PHC clinic, and a high score does not always imply compliance with Ideal Clinic standards, as each element is allocated a score to be achieved to ensure compliance.^5^ Good performance in all areas of PHC becomes crucial for rendering comprehensive healthcare services in this rural context, as in countries with limited resources, PHC clinics serve as the first and often the only point of contact with the healthcare system.^10,11^ Failure to implement and sustain the ICRM programme in rural PHC clinics will compromise the future implementation of national health insurance in South Africa and the achievement of universal health coverage. Universal health coverage is a requirement for achievement of the 2030 Agenda for Sustainable Development.^12,13,14,15,16^

The ICRM programme’s success has mostly been measured quantitatively.^5,9^ However, there is a dearth of explorative studies of the ICRM programme. Therefore, a qualitative approach was used for this study to gain an understanding of the implementation challenges and successes from the perspectives of healthcare managers and professional nurses in a specific rural subdistrict in KwaZulu-Natal. By identifying both barriers and facilitators, the researcher intended to provide valuable insights with the potential to improve the implementation of the ICRM programme for improved PHC service delivery. This qualitative inquiry serves as a valuable complement to existing quantitative studies, contributing to a comprehensive understanding of various factors affecting healthcare service quality and universal access in rural PHC clinics.^17,18^

While the implementation of the ICRM programme is typically perceived as the responsibility of nurses, it is essential to highlight that the ICRM programme manual emphasises the involvement of healthcare managers and staff at all levels. This article seeks to provide insights and guidance to healthcare professionals, particularly those in rural areas, as a means to enhance their understanding of the ICRM programme implementation.

## Research methods and design

### Study design

A qualitative case study design was used to comprehensively explore and analyse the implementation of the ICRM programme in rural PHC clinics. The case, defined as a specific functioning entity,^19,20^ was the ICRM programme implementation within the bounded system of the rural PHC clinics in the selected subdistrict.

### Study setting

The study was conducted in 7 of the 15 PHC clinics situated in a rural subdistrict in north-eastern KwaZulu-Natal. All the PHC clinics were expected to implement the ICRM programme and strive towards an Ideal Clinic status and quality care.^5^ Implementation in this study denotes the process of putting plans into action to achieve a desired rating for each of the Ideal Clinic’s 10 components. The total population of the identified sub-district was approximately 305 000, with most dependant on public PHC services as opposed to private and other healthcare options.^21^ There was a high prevalence of socioeconomic-related diseases in the region because of unemployment and food insecurity.^21^

### Study population and sampling strategy

Participants included professional nurses implementing the ICRM programme in selected PHC clinics and healthcare managers overseeing this implementation. Initially the researchers aimed to sample PHC clinics with varying distances from the district hospital and with varying client volumes using maximum variation sampling. However, because of the coronavirus pandemic (COVID-19), several PHC clinics were closed and only 7 of the 15 PHC clinics in the subdistrict agreed to participate. Four of the seven participating PHC clinics were more than 10 km from the district hospital, two were within 10 km and one was within the district hospital as a gateway PHC clinic. All PHC clinics had a medium to high client volume.

The population of professional nurses were those permanently employed at the selected PHC clinics and between the age of 22 and 65 years. The healthcare managers had to be permanently employed in the subdistrict and had to supervise the implementation of the ICRM at the selected public PHC clinics. Convenience sampling was used to sample professional nurses and healthcare managers from the seven clinics and management offices based on their availability. Professional nurses had to have at least six months of experience in the implementation of the ICRM programme. Healthcare managers had to have at least six months of experience supervising the selected PHC clinics. Data saturation, the stage where no new information was obtained, determined the final sample size and was achieved after 13 interviews. The data patterns were confirmed with another two interviews.

### Data collection

Telephonic, individual and semi-structured interviews were conducted using an interview guide. The interview guide consisted of open-ended questions on demographic information, organisational aspects of the ICRM programme, barriers, facilitators and suggestions for implementation improvement. The development of the interview questions was guided by the study objectives and literature. The interview guide was pretested to refine the questions’ clarity. Data collection occurred from June 2021 to December 2021, with telephonic interviews being used to mitigate COVID-19 risks during the lockdown in South Africa. The interviews were conducted in English because all participants were healthcare professionals who understood English. The interviews, lasting an average of 36 min, were audio-recorded to ensure accuracy. In addition, reflexive notes were taken to ensure the depth of data analysis.

### Data analysis

The researcher used Creswell’s systematic approach to qualitative data analysis consisting of six steps.^19^

- Step one: **data preparation**. The audio-recordings were transcribed by a professional transcriber. The audio-recordings were used to gain a better understanding of the phenomena under study and to validate the accuracy of the transcripts. Thereafter, transcripts were uploaded to ATLAS.ti version 9.0 for coding.
- Step two: **iterative process**. The authors gained an understanding of the data. They read the transcripts after each interview and reflected on the meaning. Notes were made on the initial ideas and patterns in the data. This process was repeated throughout the data collection, guided by the authors, allowing them to identify the credibility and depth of the data.
- Step three: **coding**. The data were coded by an independent professional coder in ATLAS.ti, using descriptive coding. The authors reflected on the categorisations and discussed the coding and categorisations with the coder.
- Step four: **categorisation**. To develop themes and subthemes. This process was guided by the study supervisors. Similar codes were merged into themes as broad categories. Descriptions were given to the themes. Subthemes were developed from the data in each theme.
- Step five: **assessment**. This assessed the consistency of the themes, subthemes and data. The data was examined to ensure the correct meanings were portrayed. The themes, subthemes and their descriptions were further refined.
- Step six: **interpretation**. Positioning the findings in the larger body of literature.

### Trustworthiness

The researcher applied Lincoln and Guba’s (1985) strategies to ensure credibility, dependability, confirmability, authenticity and transferability of the research findings.^22^ Data saturation was used to determine the sample size, ensuring comprehensiveness of the data for credibility.

To enhance dependability, the steps of the research process were followed in a structured manner and two study supervisors monitored the process. The researcher (DTM) who collected and analysed the data was working as a professional nurse in the same subdistrict but not in the clinics included in the study. Therefore, the researcher had an understanding of the context but applied bracketing throughout the data analysis process to reflect on and set aside biases.^23^ The data analysis involved the two study supervisors and an independent coder to further account for confirmability and credibility. The participants’ views were presented with supporting verbatim quotations enhancing authenticity. A description of the research setting and participants’ characteristics supports transferability and allows the reader to identify the relevance of the findings to another setting.

### Ethical considerations

Ethical clearance to conduct this study was obtained from the University of South Africa, Health Studies Higher Degrees Ethics Review Committee (No. HSHDC/984/2020) and the KwaZulu-Natal Department of Health (No. KZ_202006_025).All procedures involving human participants were carried out in accordance with the ethical standards of the institutional research committee and with the 1964 Helsinki Declaration and its later amendments or comparable ethical standards. Written informed consent was obtained from all individual participants involved in the study. Verbal consent for the audio-recording was asked just before the beginning of each telephonic interview. Interviews were conducted in privacy and all information discussed during research was not shared with anyone outside the research team. Members of the research team signed a confidentiality agreement. The data were de-identified, and codes were used in reference to participants.

## Results

### Participant characteristics

Seven professional nurses and eight healthcare managers were interviewed. The characteristics of the participants are outlined in Table 1. Seven of the eight healthcare managers were operational nurse managers overseeing the functioning of the PHC clinics, while one was a manager supervising PHC and ICRM implementation across the subdistrict. Professional nurses were unavailable at two PHC clinics, resulting in exclusive participation by healthcare managers from these two clinics.

**TABLE 1:** Participants’ characteristics.

Participant identification code	Position	Age (years)	Gender	Years of experience in PHC	Years of management experience
PN01	Professional nurse	29	Male	5	Not applicable
PN02	Professional nurse	30	Female	6	Not applicable
PN03	Professional nurse	35	Female	13	Not applicable
PN04	Professional nurse	42	Female	12	Not applicable
PN05	Professional nurse	44	Female	21	Not applicable
PN06	Professional nurse	55	Female	21	Not applicable
PN07	Professional nurse	54	Female	22	Not applicable
MAN01	Manager of PHC in the subdistrict	49	Male	15	11
MAN02	Operational nurse manager	48	Female	14	12
MAN03	Operational nurse manager	52	Male	14	11
MAN04	Operational nurse manager	61	Female	15	13
MAN05	Operational nurse manager	Not provided	Female	11	11
MAN06	Operational nurse manager	40	Female	11	5
MAN07	Operational nurse manager	40	Female	9	5
MAN08	Operational nurse manager	Not provided	Female	11	11

MAN, healthcare manager; PHC, primary healthcare; PN, professional nurse.

Participants’ ages ranged from 29 to 61 years. Twelve participants were female and three were male. The participants’ years of experience working in PHC ranged from 5 to 22 years. Six of the eight healthcare managers had 11–15 years of experience in the management of PHC and two have 5 years of experience.

### Thematic data

The analysis of the data yielded three overarching themes and eight corresponding subthemes, as detailed in Table 2.

**TABLE 2:** Overview of the themes and subthemes.

Theme	Subtheme
1. The organisation of the implementation of the ICRM programme	1.1 Regulatory framework for ICRM programme implementation 1.2 The assessment of compliance with quality standards 1.3 The rationale for the implementation of the ICRM programme
2. Barriers to the implementation of the ICRM programme	2.1 Overburdened PHC 2.2 Behavioural and organisational challenges 2.3 Resource challenges
3. Facilitators of the implementation of the ICRM programme	3.1 Stakeholder support 3.2 Teamwork and benchmarking

PHC, primary healthcare; ICRM, Ideal Clinic Realisation and Maintenance.

### The organisation of the implementation of the Ideal Clinic Realisation and Maintenance programme

Participants, in particular the professional nurses, indicated that the regulatory frameworks guide the organisation of ICRM implementation. Assessment of compliance to the Ideal Clinic quality standards is integral to the implementation of the ICRM programme. The participants demonstrated a comprehensive understanding of the organisation of the ICRM programme, showcasing their knowledge as implementers of the programme. The responses provided revealed a consistent approach to programme implementation across various PHC clinics within the subdistrict, along with the underlying reasons for its execution.

#### Regulatory framework for Ideal Clinic Realisation and Maintenance programme implementation

The participants emphasised that the implementation of the ICRM programme is reinforced by adherence to standards and guidelines in clinical practice, for example, adult primary care guidelines, HIV management guidelines, and TB and maternity guidelines. The utilisation of manuals was identified as instrumental in aiding PHC managers at all levels to ensure alignment with prescribed quality standards. The regulatory framework governing the implementation of the ICRM programme played a pivotal role in guiding activities within clinical practice carried out with the aim of attaining and maintaining Ideal Clinic status:

‘It is implemented using the two manuals the one for the ideal clinic and other one for the national core standards we should always observe when we do our work at all the clinics.’ (PN03, Female, 35 years old)

‘The ideal clinic manual guides us in the implementation of the programme and it has all the key elements to be considered in order to achieve the ideal clinic status.’ (PN06, Female, 55 years old)

#### The assessment of compliance with quality standards

Primary healthcare clinics undergo assessments conducted by various structures or teams to evaluate their adherence to standards and attainment of an Ideal Clinic status within specific assessment cycles. Participants emphasised their involvement in both implementing the programme and assessing compliance with its prescripts through formative self-assessment. Self-assessment activities present an opportunity for professional nurses to discern the nature of programme elements and prepare for the main summative assessments. The imperative to achieve and maintain Ideal Clinic status was underscored as crucial for ensuring the long-term viability of the programme in PHC clinics:

‘We are involved in implementing all the elements of the programme and we even assist the operational manager during the assessments in order to learn more about the programme since we need to be in line with what the national core standards and the programme says in order to be able [*to*] render quality services.’ (PN07, Female, 54 years old)

‘The people from management come and do the assessments in prescribed times, but our operational manager always encourage us to assess ourselves and give ourselves a score and try to improve where necessary.’ (PN05, Female, 44 years old)

#### The rationale for the implementation of the Ideal Clinic Realisation and Maintenance programme

Professional nurses, as the main implementers of the ICRM programme, indicated that the overarching goal of the programme is equitable access to high-quality healthcare for the South African population, thereby advancing the attainment of universal health coverage. The orchestrated activities within the programme’s implementation are designed to fortify PHC clinics in readiness for the national health insurance rollout. Moreover, the programme incorporates robust monitoring and evaluation mechanisms to uphold and ensure the provision of healthcare services of the highest quality:

‘To ensure that people have access to quality healthcare at the clinics and to prepare for the national health insurance and to ensure universal health coverage.’ (PN06, Female, 55 years old)

‘It is implemented to maintain the quality care and ensure that all citizens are covered with essential healthcare services in preparation for the National Health Insurance. Reduce medic-legal risks.’ (PN01, Male, 29 years old)

### Barriers to the implementation of the Ideal Clinic Realisation and Maintenance programme

The participants provided insight into the specific barriers inherent to the rural PHC context impeding the implementation of the ICRM programme and rendering quality PHC services. Healthcare managers and professional nurses found that their capacity is overstretched because of the rapidly increasing disease burden. Furthermore, behavioural and resource challenges were identified, particularly prevalent in rural PHC clinics.

#### Overburdened primary healthcare

In the rural setting, characterised by a dynamic disease burden, overburdened PHC constituted an obstacle preventing clinic staff from rendering high-quality care to clients. The prevalence of both communicable and non-communicable diseases exacerbates the strain on the already constrained human and material resources. New guidelines are regularly introduced for disease management, which places an additional burden on the staff, with participants emphasising the importance of the timely introduction of new guidelines. The timely rollout of guidelines would enable the staff as implementers to monitor service delivery, ensuring that everyone becomes familiar with the introduced guidelines and that they are correctly interpreted and applied:

‘Also when it comes to guidelines, allow the guideline to be known by staff before implementing the next one because it affects quality if you do not wait for the results of the guidelines before introducing the next one.’ (MAN01, Male, Manager in subdistrict)

‘Another thing, it will be rapidly changing guidelines in response to changing disease burden and it becomes challenging to keep up with the changes. You see every programme will come with a guideline and some guidelines may overlap.’ (MAN02, Female, Operational nurse manager)

#### Behavioural and organisational challenges

Behavioural and organisational factors also compromise the effective delivery of the ICRM programme. Challenges included non-adherence to protocols because of role confusion, poor communication, burnout and financial constraints. Burnout leads to a lack of motivation to comply with standards and could be attributable to the staff shortages and increasing patient numbers. Furthermore, staff shortages limit the opportunities and time to attend training on the ICRM programme and other quality improvement initiatives:

‘Sometimes the implementation is difficult in some elements not all of them since we are told to cut on the cost and financial constraints are always a problem. But the implementation would be much better and interesting if we have the necessary resources.’ (MAN08, Female, Operational nurse manager)

‘Demotivation on the staff’s side also proves to be a barrier and affects all other quality programmes.’ (PN05, Female, 44 years old)

‘Being short-staffed also causes staff not to attend workshops leading to lack of knowledge.’ (PN01, Male, 29 years old)

#### Resource challenges

The rural PHC setting was characterised by suboptimal clinic infrastructure, information technology challenges, insufficient supplies and a lack of essential equipment, all of which have a detrimental effect on the transition from red or orange status to green status on the Ideal Clinic dashboard. The shortage of space posed difficulties for the implementers of the ICRM programme in orchestrating and organising PHC services along delineated streams, including acute, chronic and preventative healthcare services, thus impeding proper access to services:

‘All elements are supposed to be implemented at the clinic according to the ICRM manual, but there are those that are beyond the nurses’ control, for example, infrastructure, and you find that it affects other elements like availability of streams because the streams were added, but the old clinic structures were not changed.’ (PN02, Female, 30 years old)

‘Yes, actually it’s a couple of challenges, things that I have noted. It’s a couple of them, but basically it’s lack of material resources and problems with the supply chain and maintenance.’ (MAN02, Female, Operational nurse manager)

‘Sometimes the supply chain issues come into play when it comes to procurement of services and equipment.’ (MAN06, Female, Operational nurse manager)

‘Shortage of surgical sundries, pharmaceutical supplies and equipment.’ (PN01, Male, 29 years old)

### Facilitators of the implementation of the Ideal Clinic Realisation and Maintenance programme

Participants identified key elements that facilitated the implementation of the ICRM programme, including stakeholder support and collaborative teamwork. The stakeholders are the non-governmental organisations (NGOs) and supportive subdistrict management. The PHC management, in turn, deployed strategic measures to build the capacity of its personnel, thereby ensuring the realisation of intended outcomes, including meeting community needs, and adhering to acceptable quality standards.

#### Stakeholder support

Both healthcare managers and professional nurses attested to the significance of stakeholder support as a facilitator of the implementation of the programme. Non-governmental organisations and subdistrict management provided assistance and technical guidance on aspects of the ICRM programme, thus smoothing the trajectory of its implementation. The implementation of the ICRM programme was further facilitated by community participation, as there were functional clinic committees in the PHC clinics that acted as connections between management and the community. Community involvement, in particular, empowered service recipients to provide feedback on their experiences of care, thereby contributing to the continual enhancement of healthcare service quality:

‘Consistent support and consultation from the top management in the mother hospital and support from other clinics or peers with constructive feedback and introspection on the performance of each clinic.’ (PN01, Male, 29 years old)

‘And we usually have the meetings with NGOs to talk about the areas that need improvement in our functioning as staff as that helps us to achieve the ideal clinic status it identifies the problems early.’ (PN06, Female, 55 years old)

‘Yes, I need to take the staff, the management, the community on board so that the clinic is run smoothly. Our community is also now forthcoming with suggestions, complaints, and compliments. So, the ideal clinic makes us to display the details of the operational manager for our clients to see so that there is some little bit of transparency, and so that the community can now voice out their suggestions because we encourage community involvement.’ (MAN02, Female, Operational nurse manager)

#### Teamwork and benchmarking

Both healthcare managers and professional nurses consistently identified teamwork as a facilitative factor. Additionally, benchmarking with other PHC clinics was a facilitator for achieving Ideal Clinic status. Whether involving clinical or non-clinical staff, the imperative of teamwork and support influenced the overall performance of the programme:

‘Also we support each other as clinicians from this clinic and sub-district in order to give each other tips and share important information based on experiences.’ (PN02, Female, 30 years old)

‘We capitalise on benchmarking information especially from performing clinics to assist with the stabilisation of the programme implementation and try to do those things that they are doing right.’ (MAN02, Female, Operational nurse manager)

## Discussion

The study’s findings from the perspective of managers and implementers showed that barriers to and facilitators of the implementation of the ICRM programme for quality PHC services existed on multiple levels, including individual staff member level, organisation level and system level. A key finding is the significant role played by the procedure manuals and the ICRM manual in guiding the implementation of the ICRM programme. The ICRM manual is one of the innovations of the programme, and the study supports its effectiveness in guiding professional nurses and managers at all levels, supporting standardised, quality care.^9^ However, despite the importance of these manuals, a lack of adherence was identified and could have been caused by several challenges in the rural context.

Healthcare facilities in rural areas in South Africa, typically situated in the regions that are hard to reach because of the poor conditions of the roads, frequently face shortages of essential resources necessary for efficient healthcare delivery. This situation is not unique to the rural context, as indicated by Li, Krumholz and Yip^24^ and West et al.,^25^ who state that PHC clinics are overburdened as a result of an increasing and dynamic disease burden and staff shortages. Just as in other public healthcare settings in South Africa, the lack of financial and human resources impedes effective service delivery.^26,27,28^ Infrastructural problems, including limited space in PHC clinics and insufficient supplies, affect access to and rendering of PHC services according to the expected standards for achieving Ideal Clinic status.

For effective change in PHC services and achievement of Ideal Clinic status, facility upgrades and resources need to be budgeted for at national and district levels. These change processes take time and are compromised by competing budgeting priorities in the context of the growing disease burden, which negatively affects the achievement of Ideal Clinic status within the anticipated time frames for implementation of the national health insurance following the passage of the National Health Insurance Bill in June 2023.^29^ Continued fragmentation, the increased disease burden, staff shortages and financial constraints impede rather than promote universal access to healthcare for all South African citizens, and these conditions are generally worse in rural areas.^30^ Therefore, a specific focus on enhancing the facilitators is vital.

The identified key facilitators of the implementation of the ICRM programme indicate how the programme can be supported and maintained for sustainable implementation. Participants identified facilitators such as teamwork and stakeholder support, emphasising their positive impact on service quality enhancement. Implementation science supports collaboration and teamwork as the mechanisms of change for effective implementation to improve healthcare.^31^

The pooling of resources is an additional benefit of collaboration and stakeholder support and is of particular value in this rural and under-resourced subdistrict.^32^ Collaboration and teamwork on all levels emerged as facilitators of the implementation of the ICRM, namely among the implementers in the PHC clinics, among implementers and managers, benchmarking with other PHC facilities in the subdistrict and support from stakeholders, including NGOs and higher levels of management. Staff involvement in assessment within the PHC clinics, particularly formative self-assessment, appears to facilitate the implementation and maintenance of the ICRM programme. This process encourages the co-design of improvement strategies, fostering a collaborative approach and commitment to improvements and change.^33^

Stakeholder support from NGOs took the form of supplies, staff development workshops, provision of educational materials during community awareness campaigns, supplementation of human resources to assist with meeting the targets in the priority programmes, improved quality patient care and improved access to counselling services. Non-governmental organisations’ support is specifically important to achieve 100% of the vital elements and 70% of the essential elements of an Ideal Clinic. The population’s health can be adversely affected if vital elements are not achieved, and patient care can be indirectly affected if essential elements such as process and structural elements are not achieved.^9^ This makes NGO support an important strategy for increasing the effectiveness of the ICRM programme and therefore strengthening the healthcare system. This mirrors the effectiveness of NGOs in supporting other PHC programmes in South Africa.^26,34^ Active engagement, clear communication with and feedback from NGOs are required for mutual learning that benefits the NGO, the healthcare professional and the client.^34,35^

Another key facilitator of the maintenance of quality PHC service standards is community participation, facilitated by functional clinic committees. These clinic committees give service recipients (the population) the opportunity to provide feedback and allow for responsive adaptations of services. Sharing information raises awareness among service recipients.^36^

The recommendations for improving and maintaining the implementation of the ICRM programme in rural KwaZulu-Natal can be derived from the key findings. Implementation strategies are most effective when they are intended to overcome specific barriers and maintain or enhance the facilitators.^37,38^ Furthermore, combining implementation strategies directed at different levels of implementation is more effective than single, discrete strategies.^39^

On the individual level, the professional nurses as the implementers of the ICRM programme should be trained to enhance their knowledge of the standards of the ICRM programme and to stay abreast of new guidelines. Training can facilitate faster integration of new clinical practice guidelines. Training is also essential to orientate new staff, and refresher courses are necessary for effective healthcare service delivery. Achieving a common level of understanding among staff is required for teamwork and a known facilitator of the implementation of the ICRM programme.^31^ Effective sharing of information, particularly regarding the proper application of clinical guidelines, plays an important role in fostering collaboration within the team.^40,41^

Teamwork is important for the effective implementation of interventions, including the implementation of the ICRM programme. Therefore, strategies for motivating staff and managing burnout are imperative to achieve commitment to programme implementation. However, burnout and job dissatisfaction are influenced not only by individual factors but also by organisational factors such as staff shortages and a high workload.^42,43,44^

Several organisational factors affecting the implementation of the ICRM programme, including a lack of staff and money, are not unique but are emphasised more in the rural PHC context across the country and mainly in the South African rural public healthcare context. These contextual factors affect the quality of healthcare services. Rural PHC clinics face an increased number of clients because of an increasing population, disease burden and staff shortages.^44,45^ The budgets for resources and staff should accommodate the increasing client volumes at rural PHC clinics. High staff turnover and an inability to recruit skilled personnel further exacerbated the situation.^21,46^ These issues led to burnout among PHC clinic staff, who faced overwhelming workloads and missed training opportunities because of staff shortages. High patient numbers and increased workload sometimes led to nurses not being able to properly explain conditions and treatment regimens to the clients during consultation. The rising disease burden intensified the demand for pharmaceutical supplies, with supply already constrained in South Africa, especially in rural healthcare facilities.^35,47^

Some barriers at the organisational level may be difficult to overcome because of budget constraints or government decisions. However, other barriers can be addressed in a shorter time through, for example, avoiding the simultaneous implementation of too many new guidelines at PHC clinics. Participants appeared to prefer a more gradual introduction of change, especially when more than one intervention was introduced. This recommendation aligns with the literature, which suggests that implementing changes to one specific aspect at a time is more effective than introducing changes to multiple aspects simultaneously.^48,49^

With regard to system-level challenges, the responsibilities of relevant stakeholders, such as government, in the healthcare sector include infrastructural overhaul to facilitate the provision of care according to the expected standard relating to the healthcare streams in dedicated areas. Some of the clinic buildings in the study area are old, making it difficult to sort clients and organise care according to necessary PHC streams.^42,50^ Furthermore, financial support and improved communication are vital for positive health outcomes, as communication is one of the elements of the ICRM programme, and it is essential to maintain communication to facilitate service delivery and improve health outcomes.

Overall, the findings suggest a need for specific support in order to maintain and sustain the implementation of the ICRM programme in rural KwaZulu-Natal PHC clinics. Similar support is needed at system level. The study suggests areas that can be improved over both the short and long term.

### Limitations of the study

As the study was conducted during the COVID-19 lockdown, problems relating to participants’ accessibility were experienced and convenience sampling was used. Despite this, data saturation was achieved, namely common patterns were identified, and no new information was identified. The study’s findings are rooted in the specific context of the rural subdistrict and certain factors may be unique. Furthermore, implementation may vary across PHC clinics limiting the transferability of the findings. Despite the context-specific findings, several barriers and facilitators are common in various PHC contexts in South Africa, including resource restrictions, an overburdened PHC system, staff shortages, and the need for teamwork and stakeholder support. Researchers and practitioners should carefully consider contextual differences when using this study’s findings, and further explorative case studies may be needed to identify similarities and differences in diverse contexts.

### Implications of the study

This study contributed to the body of knowledge and specifically provides an understanding of the barriers and facilitator that influences implementation of the ICRM programme in rural PHC clinics from the perspective of the implementers. By understanding these factors, policymakers, healthcare managers and administrators can develop more effective and targeted strategies to enhance the implementation of the ICRM programme to improve quality of healthcare services in rural settings and set the stage for implementing the national health insurance.

## Conclusion

The study aimed to explore the perspectives on the barriers and facilitators of implementing the ICRM programme at PHC clinics in a specific subdistrict in rural KwaZulu-Natal. The findings highlighted the complexities of implementing the ICRM programme in the PHC clinics, and barriers included the strain on clinics because of high demand for PHC services, staff shortages that lead to burnout, limitations posed by ageing infrastructures and a lack of essential resources. The existing facilitators were identified and included the support from NGOs and management, involvement of the recipients of care, teamwork, involvement of implementers in the assessments and benchmarking with other PHC clinics. If challenges are mitigated and supportive factors leveraged, the potential for sustained implementation of the ICRM programme and improved healthcare delivery can be realised, benefiting both healthcare providers and recipients alike.
